# Sex body: A nest of protein mixture

**DOI:** 10.3389/fcell.2023.1165745

**Published:** 2023-04-14

**Authors:** Miao Li

**Affiliations:** Department of Obstetrics and Gynecology, Shanghai First Maternity and Infant Hospital, School of Medicine, Tongji University, Shanghai, China

**Keywords:** meiosis, sex body, MSCI, DNA damage response, epigenetic modifications

## Abstract

During the pachytene stage in mammalian meiosis, the X and Y chromosomes remain largely unsynapsed outside the pseudoautosomal region, while autosomes are fully synapsed. Then, the sex chromosomes are compartmentalized into a “sex body” in the nucleus and are subjected to meiotic sex chromosome inactivation (MSCI). For decades, the formation and functioning of the sex body and MSCI have been subjects worth exploring. Notably, a series of proteins have been reported to be located on the sex body area and inferred to play an essential role in MSCI; however, the proteins that are actually located in this area and how these proteins promote sex body formation and establish MSCI remain unclear. Collectively, the DNA damage response factors, downstream fanconi anemia proteins, and other canonical repressive histone modifications have been reported to be associated with the sex body. Here, this study reviews the factors located on the sex body area and tries to provide new insights into studying this mysterious domain.

## Introduction

Mammalian meiosis is a complex and continuous differentiation process to generate haploid sperm cells in males. Programmed formation and repair of DNA double-strand breaks (DSBs) catalyzed by the topoisomerase-like protein SPO11 are the basis for the dynamic chromosomal behavior during meiosis prophase I (Keeney et al., 1997). Once produced, the DSBs are protected by the heterotrimeric RPA (replication protein A) complex, followed by the loading of the RAD51 recombinase and DNA meiotic recombinase 1 (DMC1), thus enabling strand invasion of the duplex DNA for homolog pairing and recombination (Wold, 1997; Yoshida et al., 1998; Cloud et al., 2012). At the pachytene stage, autosomes gradually complete the synapsis and DSB repair, while the heterologous X and Y chromosomes remain unsynapsed, and they only stably pair at a small homologous region called the pseudoautosomal region (PAR) and are subject to the formation of a specialized structure called the XY body or sex body (Burgoyne et al., 2009; Handel and Schimenti, 2010). Along with the sex body formation, the extensive asynapsis triggers transcriptional silencing–meiotic sex chromosome inactivation (MSCI) (Turner, 2007; Turner, 2015). The formation of the sex body and establishment of MSCI are required for the successful completion of spermatogenesis (Royo et al., 2010; Turner, 2015). These two events are tightly associated and may have a reciprocal causation relationship. The formation, maintenance, and disassembly of the sex body are dynamic processes of the meiosis prophase I stage; it is important to know what factors are located on the sex body area, either dependent on or independent of the sex body formation. In fact, the sex body contains X and Y chromosomes and includes axes, loops, and other liquid-like protein mixtures. Interestingly, the localizations of these proteins are of three types: restricted on the axes, diffused exactly in the whole sex body area, or of both which usually represents a dynamic process, spreading from the axes to loops. Is the sex body simply a repository for these proteins or do these proteins shape the sex body? This review elucidates the proteins that are located on the sex chromosome axes or sex body areas and tries to find new insights into studying the sex body.

## DDR proteins

As mentioned, the DNA damage response (DDR) proteins are thought to be the important machinery in regulating the MSCI and sex body formation, and they usually function in two key steps. First, the asynapsis is detected by “sensors,” which are localized to the axial elements. Subsequently, “effectors” spread over the chromatin loops and cause gene silencing and sex body configuration. HORMAD1/2 (HORMA domain-containing protein 1/2) are presented and restricted on the unsynapsed axes of the X and Y chromosomes during the pachytene stage, which are essential for DSB formation and synapsis (Wojtasz et al., 2009; Daniel et al., 2011). Without HORMAD1/2, the DDR signals are inhibited on the XY chromosomes, thus HORMAD1/2 are upstream of DDR (Wojtasz et al., 2009; Daniel et al., 2011). BRCA1 (breast cancer 1), an important DDR protein, accumulates on the axes of the sex chromosomes and is proposed to recruit the ATR (ataxia telangiectasia and Rad3-related) kinase to phosphorylate H2AX at Ser139 termed γH2AX on a single chromosome axis (Fernandez-Capetillo et al., 2003; Turner et al., 2004; Royo et al., 2013; Broering et al., 2014). At the early pachytene stage, ATR is only enriched on the axis together with its activator protein TOPBP1 (topoisomerase II-binding protein 1). Following the binding of MDC1 (mediator of DNA damage checkpoint protein 1) to γH2AX, TOPBP1-ATR is further activated and confined to the sex body and promotes the γH2AX signal spread to the protruding loops of the chromatin at the mid-pachytene stage (Ichijima et al., 2011; ElInati et al., 2017). BRCA1 is a primary component of four complexes: RAP80-containing BRCA1-A, BACH1-harboring BRCA1-B, the CtIP-holding BRCA1-C complex, and the BRCA1/PALB2 (also known as FANCN)/BRCA2 complex in the somatic cells (Her et al., 2016). The elements of the BRCA1-A complex, namely, CCDC98, RAP80 (receptor-associated protein 80), and MERIT40 (mediator of Rap80 interactions and targeting 40 kd) are observed on the axes of the sex chromosomes to facilitate the loading of RAD51 to the DSB sites (Liu et al., 2007; Feng et al., 2009; Wu et al., 2012; Lu et al., 2013). CtIP from the BRCA-C complex is specifically localized to the unsynapsed axes of the sex body, while BRCA1-B is not observed on the sex chromosomes (Yu and Chen, 2004; Sartori et al., 2007). The BRCA1/PALB2 (also known as FANCN)/BRCA2 complex is discussed below. The DDR proteins are reported to be potential phase-separating proteins which have a high content of IDR (intrinsically disordered regions) and might regulate sex body formation by driving phase separation (Xu and Qiao, 2021).

## RNF8/RAD18/53BP1 pathway and ubiquitin modifications

RNF8 has been shown to participate in the DNA damage response by interacting with MDC1 in somatic cells (Huen et al., 2007). During meiosis, RNF8 is essential for protein ubiquitination in the sex body, which is not important for MSCI but indispensable for the replacement of histones by protamine during spermiogenesis (Lu et al., 2010). The downstream proteins of RNF8, RAD18 (RAD18 E3 ubiquitin protein ligase), and 53BP1(transformation-related protein 53-binding protein 1) are localized to the entire sex chromosomes (van der Laan et al., 2004). However, it has been reported that either RAD18 or 53BP1 does not affect fertility in mice (Ward et al., 2003; Sun et al., 2009). Ubiquitinated proteins, detected by the monoclonal antibody FK2 (ub-FK2), and RNF8-dependent ubiquitinated H2A (ub-H2A, detected by monoclonal antibody E6C5) are localized on the sex body area (Lu et al., 2010). K48- and K63-linked poly-ubiquitins are enriched in the XY body (Lu et al., 2010). The role of ubiquitination around the XY body requires further investigation since suppression of ubiquitination shows no effect on the meiosis process. SUMOylation (small ubiquitin-like modifier) is another ubiquitination-like protein modification at the DNA damage sites. SUMO1 and SUMO2/3 are enriched in the XY body over the sex chromosomes (La Salle et al., 2008). The SCML2 (sex comb on midleg-like 2)–USP7 (ubiquitin-specific peptidase 7) pathway could regulate ubiquitination on sex body areas (Luo et al., 2015). SCML2 is an X chromosome–encoded polycomb protein, which is associated with γH2AX and localized to the XY body in spermatocytes (Hasegawa et al., 2015; Luo et al., 2015). The loss of SCML2 in mice causes defective spermatogenesis, resulting in sharply reduced sperm production (Hasegawa et al., 2015; Luo et al., 2015). SCML2 recruits deubiquitinase USP7 to the XY body in spermatocytes. In the absence of SCML2, USP7 fails to accumulate on the XY body, whereas ub-H2A dramatically augments in the XY chromatin (Luo et al., 2015). Neither ubiquitination nor SUMOylation is directly involved in sex body formation, which is more a result of sex chromosome compartmentalization.

## Other DNA repair proteins: Fanconi anemia pathway

Fanconi anemia (FA) is a genetic disease associated with bone marrow failure, increased cancer susceptibility, and severe germline defects (Soulier, 2011). The FA core complex is a multicomponent ubiquitin ligase, comprising the catalytic component (FANC-B, FANC-L, FAAP100), substrate adaptor (FANC-C, FANC-E, FANC-F), and FANC-A, FANC-G, FANC-M, and FAAP20, which are localized at the sites of DSBs (Benitez et al., 2018). FANC-M accumulation on the sex chromosome axis begins in the early pachytene stage and spreads on to the entire domain of the X and Y chromosomes during the early diplotene stage (Alavattam et al., 2016). FANC-M knockout mice have smaller testis and reduced follicles (Bakker et al., 2009). FANC-B, another FA core protein, accumulates on the sex chromosomes in the early pachytene stage which is essential for male fertility and regulates H3K9me2 and H3K9me3 on the sex chromosomes during meiosis (Kato et al., 2015). FA core complex then catalyzes the monoubiquitination of the FANCD2-FANCI heterodimer (Smogorzewska et al., 2007). FANCI signals spread throughout the entire XY domain in the early diplotene stage and regulate H3K9me2 and H3K4me2 on the sex chromosome which is essential for male fertility without affecting the sex body formation (Xu et al., 2021). The FANCD2 foci are colocalized with RAD51(FANCR) on the DSB sites on both the autosome and sex chromosome axes during meiosis (Alavattam et al., 2016). In addition, FANCD2 regulates H3K4me2 (H3K4 demethylation) and H3K9me3 (H3K9 accumulation) on the XY chromatin together with RNF8 (Alavattam et al., 2016). The other two FA proteins BRCA2 (FANCD1) and SLX4 (FANCP) also located on the sex chromosomes dependent on the DDR and RNF8 pathways. The localizations of BRCA2 in meiotic spermatocytes on the sex chromosomes or sex body areas are not clear yet because of the lack of an ideal BRCA2 antibody (Chen et al., 1998; Alavattam et al., 2016). SLX4, a structure-specific endonuclease, is involved in the repair of various DNA lesions, progressively increasing in intensity as it spreads through the XY chromatin during the pachytene stages, as seen in SLX4 knockout mice with reduced fertility (Holloway et al., 2011; Alavattam et al., 2016). Taken together, FA proteins with MDC1 and RNF8 might govern the behaviors of sex chromosomes by regulating DDR and epigenetic programming during meiosis.

## Heterochromatin-related proteins and modifications

Following DDR, a histone H3-lysine-9 methyltransferase SETDB1 (SET domain bifurcated histone lysine methyltransferase 1) was recruited to the sex chromosomes, which is responsible for H3K9me3 modifications on the X chromosome and essential for sex chromosome remodeling and silencing (Hirota et al., 2018). TRIM28 (tripartite motif-containing 28) possibly bridged DDR to SETDB1 and regulated transcription, and was also located around the sex chromosomes during the pachytene stage (Hirota et al., 2018). TRIM28-SETDB1 probably promote gene silencing through depositing suppressed histone modifications on the sex chromosomes. Histone methyltransferase SUV39H2 (suppressor of variegation 3-9 homolog 2) accumulates on the sex body region in pachytene spermatocytes to modulate heterochromatin (O'Carroll et al., 2000). Heterochromatin protein 1 (HP1), which binds to H3K9me3, is shown to be related to chromatin condensation and transcription regulation. Two isoforms of the HP1 proteins, HP1β and HP1γ, decorate the entire sex body at the late pachytene stage in spermatocytes, indicating their potential roles in condensing and silencing the sex chromosomes (Metzler-Guillemain et al., 2003). MacroH2A1, a histone variant and CHD3/4 (chromodomain helicase DNA-binding protein 3/4), specifically accumulate on the X-centromeric end and PAR of meiotic sex chromosomes (Turner et al., 2001; Broering et al., 2014). On the other hand, the active modifications of H4K20 monomethylation (H4K20me1 and H3K4me2) and H3K27ac (H3K27 acetylation) accumulate on the sex chromosomes in the late pachytene stage and could join the activation of gene expression in subsequent spermatogenesis (Sin and Namekawa, 2013; Adams et al., 2018). Also, this might be the reason why chromatin accessibility in the XY chromosomes is more open during MSCI when gene silencing occurs (Patel et al., 2019).

## PAR-related proteins and recombination proteins

The formation of DSBs on PAR is the driving force for pairing and crossing over between X and Y chromosomes and the subsequent sex body formation (Acquaviva et al., 2020).

REC114 (REC114 meiotic recombination protein), ANKRD31 (ankyrin repeat domain 31), IHO1 (interactor of HORMAD1), MEI1 (meiotic double-stranded break formation protein 1), and MEI4 (meiotic double-stranded break formation protein 4) located on the PAR are essential for efficient DSB formation (Acquaviva et al., 2020). The persistent DSBs on the sex chromosomes are recognized by recombination proteins (Baudat et al., 2013). RPA binds to the ssDNA end and facilitates the exchange of RAD51/DMC1 into the ssDNA (Baudat et al., 2013; Zickler and Kleckner, 2015). The recombination proteins are located on DSB sites, therefore RPA2, RAD51/DMC1, MSH4/5 (mutS homolog 4/5), and MLH3 (mutL homolog 3) are detected on the sex chromosome axes (Abe et al., 2019). These proteins are recruited to the sex chromosomes before the sex body formation, and their locations are independent of sex chromosomal behavior. Thus, these proteins seem to not take part in sex body establishment directly.

## Other proteins

XLR6 (X-chromosome linked, lymphocyte regulated) accumulates on the sex body in the early pachytene to mid-pachytene spermatocytes and is re-localized to the nucleolus during late pachytene, although XLR6 does not seem to be essential for fertility (Tsutsumi et al., 2011; Li et al., 2015). SETX (senataxin) is concentrated on the sex chromosomes, and *Setx* ^*−/−*^ spermatocytes are arrested at the pachytene stage (Becherel et al., 2013; Yeo et al., 2015). MRNIP (MRN complex-interacting protein) is condensed to one large droplet-like assembly which partially overlaps the sex body chromatin (Kazi et al., 2022). *Mrnip* ^*−/−*^ spermatocytes display defective sex body formation and MSCI (KAZI ET AL., 2022). AGO4 (argonaute family member 4) functions in microRNA regulation to regulate MSCI which is located on the XY body area (Modzelewski et al., 2012). MAPS (male pachynema-specific protein) concentrated on the XY body regulates XY body formation and MSCI (Li et al., 2021).

## Conclusion

During meiosis in male mice, the sex chromosomes are reorganized to be a non-membrane organelle, which is distinguished as a special area isolated from other autosomes observed using electron microscopy (Solari, 1974). Although the molecular mechanism of the sex body and MSCI have been studied, it is not yet clear how the sex body and gene repression are mediated. Here, this review summarizes several pathways and proteins that accumulate on the sex chromosome axes or sex body areas and play potential roles in the sex body. Generally speaking, at the initiation of MSCI, DDR proteins are recruited to the X and Y chromosome axes and loops in response to asynapsis, downstream of the DDR, and unique epigenetic modifications are shaped leading to the formation of the sex body (Turner, 2015; Abe et al., 2019). Many other proteins take part in this process, which includes the FA pathway, RNF8-dependent pathway, and heterochromatin-related modifications (Alavattam et al., 2016). Recent evidence suggests that MSCI and sex body formation may be driven by phase separation, a physical process that governs the formation of membraneless organelles and other biomolecular condensates (Alavattam et al., 2021; Xu and Qiao, 2021). However, how these sex body–localized proteins drive the sex body formation step-by-step and how MSCI maintained in spermatocytes remain unclear. To find out more proteins and elucidate the protein interaction relationship could pave the way for understanding the mysterious domain.
